# Single-Cell Transcriptomic Profiling of Peripheral Blood in a Patient with Progressive Thyroid Eye Disease Following Total Thyroidectomy

**DOI:** 10.3390/genes17091143

**Published:** 2026-09-18

**Authors:** Ainura Mussakulova, Zarina Zhalgasbaeva, Vyacheslav Korobeynikov, Gulnur Zhunussova

**Affiliations:** 1 Laboratory of Molecular Genetics, Institute of Genetics and Physiology, Almaty 050060, Kazakhstan; 2 Faculty of Medicine and Health Care, Al-Farabi Kazakh National University, Almaty 050040, Kazakhstan; 3 Faculty of Biology and Biotechnology, Al-Farabi Kazakh National University, Almaty 050040, Kazakhstan; 4 Cancer Research Institute, Tomsk National Research Medical Center, Russian Academy of Sciences, Tomsk 634009, Russia

**Keywords:** thyroid eye disease, Graves’ orbitopathy, total thyroidectomy, single-cell RNA sequencing, peripheral blood mononuclear cells, *CXCR4*, immunometabolism

## Abstract

Thyroid eye disease (TED) is an autoimmune inflammatory disorder of the orbit closely associated with thyroid dysfunction. Total thyroidectomy is traditionally expected to attenuate the autoimmune cascade, yet some patients continue to progress after surgery, which challenges this paradigm and points to a potentially autonomous orbital immune response. To investigate this, we performed single-cell RNA sequencing (scRNA-seq) of peripheral blood mononuclear cells (PBMCs) from a 47-year-old woman with severe, glucocorticoid-refractory TED, four years after total thyroidectomy, and compared her immune landscape with that of an age- and sex-matched healthy control. The patient’s peripheral blood showed a clear shift toward an active, stress-associated inflammatory state: pro-inflammatory mediators, immediate-early response transcription factors (*FOS*, *JUNB*, *FOSB*, *TNFAIP3*), and survival-related genes (*CXCR4*, *DDIT4*) were all upregulated. Gene Set Enrichment Analysis (GSEA) pointed to different mechanisms across cell types—myeloid cells were enriched for IL-6/JAK/STAT3 and TNF-α signaling, activated T cells for oxidative phosphorylation and mTORC1 signaling—while B lymphocytes and NK cells showed the opposite pattern, with reduced inflammatory and metabolic activity. One possible explanation is a “tissue-drainage” effect, where the most pathogenic lymphocytes leave the bloodstream and migrate into the inflamed orbital tissue along chemokine gradients. Together, these findings point to the *CXCR4*/*CXCL12* axis and cellular stress pathways as candidate therapeutic targets, though as a single-case observation this remains preliminary and requires validation in larger cohorts, at the protein level, and in functional in vitro/in vivo studies.

## 1. Introduction

Thyroid eye disease (TED) is a complex autoimmune inflammatory disorder characterized by the involvement of all orbital structures, including orbital adipose tissue, extraocular muscles, connective tissue, and the vascular bed, and is closely associated with thyroid dysfunction. The annual incidence of TED averages 16 cases per 100,000 among women and 2.9 cases per 100,000 among men [1]. According to global literature, TED develops in approximately 40% of patients diagnosed with Graves’ disease, whereas the remaining patient cohort exhibits no clinical manifestations of the ocular condition [2].

Within the spectrum of thyroid pathology associations, TED is linked to Graves’ disease in 80% of cases, and to autoimmune thyroiditis in euthyroid or hypothyroid patients in 10% of cases. The remaining 10% of cases develop against a background of normal thyroid hormone and thyroid-stimulating hormone (TSH) levels, without a history of clinically manifest autoimmune thyroid disease [3]. Severe, decompensated forms of TED affect 3–5% of patients [2], with up to 20–35% of individuals presenting with moderate-to-severe disease courses demonstrating resistance to first-line glucocorticosteroid therapy. Furthermore, 10–15% of patients experience disease reactivation following a brief clinical improvement upon the discontinuation of hormonal treatment [4].

Current therapeutic modalities for TED encompass systemic and pulse glucocorticosteroid therapy, orbital radiotherapy, surgical intervention, and immunosuppressive treatment [5]. However, these approaches exhibit limited efficacy, induce pronounced adverse effects, and frequently fail to halt disease progression. In particular, corticosteroid monotherapy exerts an insufficient impact on the progression of exophthalmos and fibrotic remodeling within orbital tissues, often necessitating the deployment of combined therapeutic strategies [5,6]. These therapeutic limitations highlight the need for a better understanding of the immune mechanisms that contribute to persistent or progressive TED.

It is hypothesized that total thyroidectomy leads to a reduction in thyroid-stimulating hormone receptor antibody titers and a subsequent attenuation of the autoimmune cascade. Nonetheless, clinical study outcomes demonstrate that this assumption is not always validated in practice. Investigators emphasize the critical need for standardized trials evaluating the clinical outcomes of TED following the surgical management of Graves’ disease [7,8]. Moreover, several publications report the de novo development of TED after thyroid gland removal, as well as the recurrence or progression of pre-existing ophthalmopathy [9,10,11].

Beyond the cellular and cytokine-signaling mechanisms described above, genetic susceptibility factors have also been investigated in TED. In a case control study of Kazakhstani patients with Graves’ disease, our group previously reported that the CC genotype of the IL1F10 (IL-38) rs3811058 polymorphism was significantly associated with an increased risk of TED among Caucasian patients, whereas no significant genotype or allele associations were found for the IL17F rs9463772 polymorphism, highlighting the population and ethnicity specific contribution of interleukin gene variants to TED susceptibility [12].

Consequently, the pathophysiology of thyroid eye disease remains a subject of intensive research aimed at identifying novel, targeted, minimally invasive, and more effective therapeutic methods. In recent years, single-cell analysis, specifically single-cell RNA sequencing (scRNA-seq) has attracted significant interest, as it enables the investigation of gene expression at the individual cell level rather than within an averaged bulk tissue cell population. This approach provides an unparalleled opportunity to uncover cellular heterogeneity, rare subpopulations, functional cell states, and intercellular communication networks.

To date, a limited number of single-cell studies of TED have been published [13,14,15,16,17], most of which have focused on local orbital tissues obtained during surgical procedures. Although these studies provide important insights into the local orbital microenvironment, they provide limited information about the systemic immune compartment and circulating immune cells in patients with persistent or progressive disease. Data on the single-cell transcriptional profiling of peripheral blood in patients with TED, particularly in the setting of disease progression following total thyroidectomy, remain limited. Unlike orbital tissue, which reflects the local inflammatory microenvironment, peripheral blood provides access to circulating immune cells and may help characterize systemic immune activation and cellular states associated with disease activity. Therefore, profiling peripheral blood mononuclear cells (PBMCs) in a patient with progressive TED despite total thyroidectomy may provide preliminary insight into systemic immune alterations that persist after removal of the thyroid gland and may contribute to ongoing disease activity.

In this study, we performed scRNA-seq of peripheral blood mononuclear cells from a patient with severe, glucocorticoid-refractory TED who experienced progressive disease four years after total thyroidectomy and compared the resulting immune-cell landscape with that of an age- and sex-matched healthy control. We aimed to characterize cell-type-specific transcriptional alterations and identify immune and molecular pathways potentially associated with persistent disease activity after thyroidectomy.

## 2. Materials and Methods

### 2.1. Consent and Clinical Characteristics

The study included a 47-year-old female patient presenting with a severe, active course of thyroid eye disease secondary to autoimmune thyroid disease, following a total thyroidectomy performed in 2022. A sex- and age-matched healthy individual without thyroid or autoimmune pathology was included as a control donor. Peripheral venous blood samples were collected after overnight fasting into ethylenediaminetetraacetic acid (EDTA) tubes. The study was conducted in strict accordance with the declaration of Helsinki and approved by the local ethics committee of the Institute of Genetics and Physiology of the Science Committee of the Ministry of Science and Higher Education of the Republic of Kazakhstan (protocol No. 3, approved on 19 September 2024). Written informed consent for the study and subsequent publication of the data was obtained from all participants.

### 2.2. Isolation of Peripheral Blood Mononuclear Cells (PBMCs)

Peripheral blood mononuclear cells (PBMCs) were isolated via density gradient centrifugation using Ficoll-Paque. Following centrifugation, the mononuclear cell interphase layer was harvested and washed twice with phosphate-buffered saline (PBS) at 400 g for 10 min to eliminate residual plasma. The resulting cell pellets were resuspended in PBS, and cell concentration and viability were assessed using trypan blue staining. Only samples demonstrating a cell viability of greater than 80% were captured for downstream single-cell processing.

### 2.3. Single-Cell RNA Library Construction

Single-cell RNA-seq libraries were prepared for 3′ single-cell RNA sequencing using the DNBelab C Series Single-Cell RNA Library Preparation Set V3.0 (MGI, Shenzhen, China) on a DNBelab C-TaiM 4 single-cell droplet generator (MGI, China), following the manufacturer’s standard protocol.

### 2.4. High-Throughput Sequencing

The prepared single-cell RNA libraries were sequenced on the DNBSEQ-G400 platform (MGI, China) in a paired-end mode with the following read configuration: 47 cycles for Read 1 (incorporating cell barcode and UMI), 100 cycles for Read 2 (transcript read), and 10 cycles for the index read.

### 2.5. Quality Control and Bioinformatics Data Processing

Primary processing of raw sequencing data, including demultiplexing, read alignment to the human reference genome (GRCh38), cell calling, and gene expression matrix generation, was performed using the dnbc4tools software pipeline (version 3.0). This workflow executed standard quality assessment, sequence alignment, and droplet-based cell identifier extraction.

Downstream bioinformatic and statistical analyses were performed in the R environment using the Seurat package (version 5.5.0.9000). Cells were subjected to strict quality control (QC) filtering optimized for each sample to systematically purge low-quality events, empty droplets, and multicellular artifacts, based on the number of detected genes per cell (nFeature_RNA), the total transcript count per cell as measured by unique molecular identifiers (nCount_RNA), and the mitochondrial read fraction (percent.mt). For the TED patient dataset, cells were retained based on the thresholds: nFeature_RNA < 6000, nCount_RNA < 30,000, and a mitochondrial read fraction (percent.mt) < 10%. For the healthy control dataset, the QC boundaries were defined as: nFeature_RNA < 5000, nCount_RNA < 30,000, and percent.mt < 5%. Quality-control thresholds for nFeature_RNA were determined empirically based on the distribution of this metric within each sample group, visualized using violin plots. The TED patient sample exhibited a distribution shifted toward higher values of detected genes per cell; accordingly, the upper threshold was set at 6000 for the TED sample and 5000 for the healthy control. Both thresholds fall within the commonly accepted range (200–6000) and were further verified by visual inspection of the distributions to retain the maximum number of high-quality cells in each group while avoiding the loss of biologically relevant populations. Potential homotypic and heterotypic doublets were computationally predicted and eliminated using the scDblFinder package (version 1.16.0).

Data normalization, scaling, identification of highly variable genes, and principal component analysis (PCA) were performed using standard functions integrated into the Seurat package. To correct for potential batch effects between the patient and control samples, dataset integration was performed using the Harmony algorithm on the PCA embedding. Uniform manifold approximation and projection (UMAP) dimensional reduction and clustering were subsequently performed on the Harmony-integrated embedding. Differentially expressed genes (DEGs) between the patient and control groups were calculated using the FindMarkers function in Seurat, applying an adjusted *p*-value threshold < 0.05 (with Benjamini–Hochberg correction) and a Log2 Fold Change threshold > 0.50 to guarantee stringent downstream biological significance. Functional pathway enrichment analysis was performed via Gene Set Enrichment Analysis (GSEA) using the fgsea package (version 1.28.0) in R. The analysis utilized the Molecular Signatures Database (MSigDB) Hallmark gene set collection, with enrichment significance evaluated at an adjusted *p*-value < 0.05 alongside an absolute Normalized Enrichment Score (|NES|) threshold > 1.5 to exclude weak or trivial enrichment events across the immune cell subpopulations.

## 3. Results

### 3.1. Clinical Case Presentation

**Figure 1:**
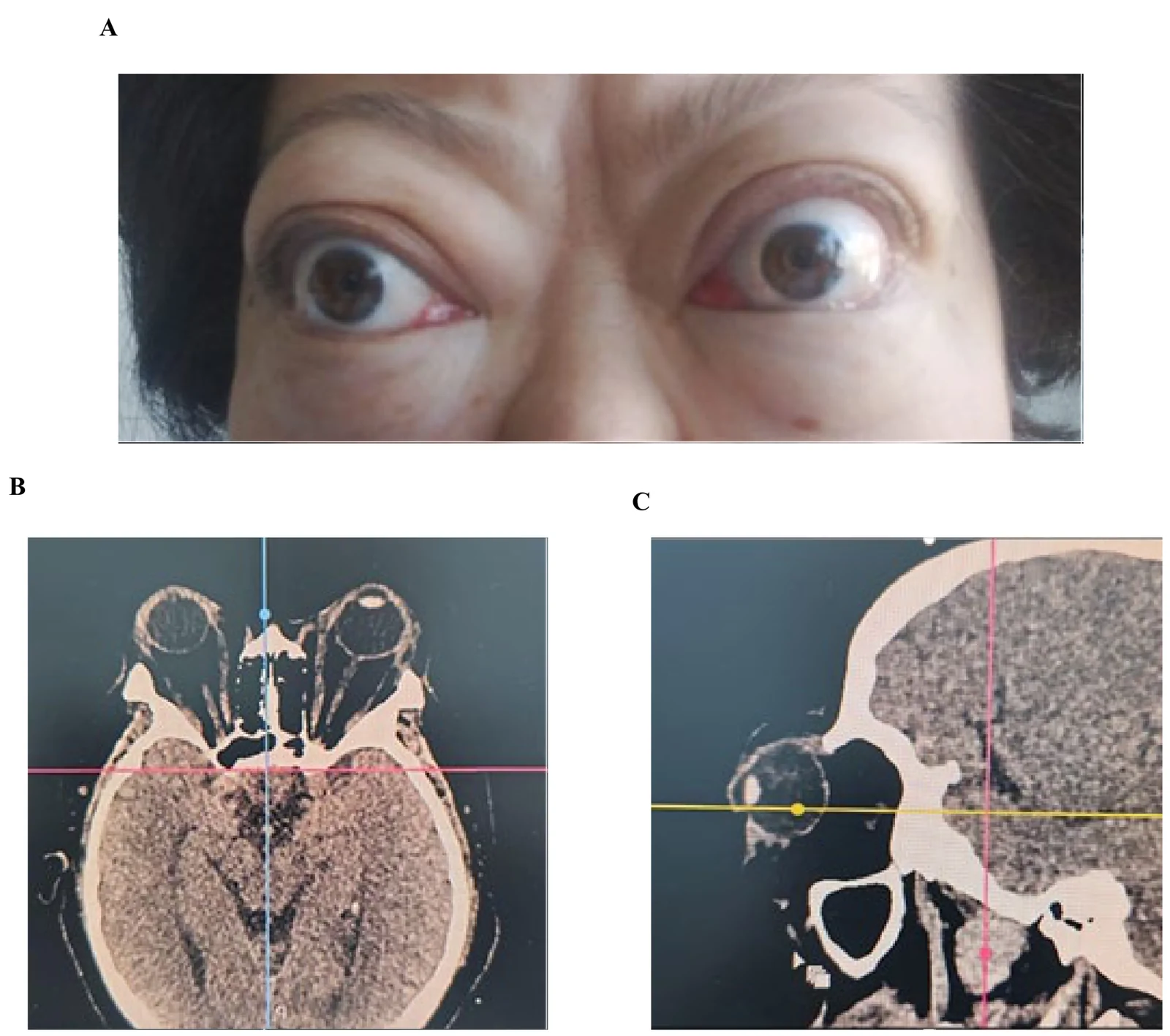
Clinical and imaging presentation of the patient with severe, active, and refractory thyroid eye disease (TED). (**A**) Clinical photograph of the patient’s ocular region demonstrating advanced bilateral exophthalmos, pronounced upper eyelid retraction, periorbital edema, and conjunctival/caruncular injection. (**B**) Axial and (**C**) sagittal computed tomography (CT) scans of the orbits, showing severe bilateral exophthalmos, significant hypertrophy of the extraocular muscles (predominantly the medial recti), marked expansion of retrobulbar orbital fat, and narrowing of the retrobulbar space. These findings collectively highlight active, chronic inflammatory and fibrotic remodeling within the retrobulbar compartment.

A 47-year-old female patient presented to the Kazakh Research Institute of Eye Diseases (Almaty, Republic of Kazakhstan) with a clinical manifestation of severe, progressive thyroid eye disease (TED) in its active phase, characterized by edematous exophthalmos at the stage of sub compensation. At the time of examination, a profound decompensation of the autoimmune inflammatory cascade within the orbital tissues was observed, as evidenced by a comprehensive set of objective clinical, functional, and imaging parameters (Figure 1; Table 1).

The patient’s medical history revealed that she was diagnosed with Graves’ disease (diffuse toxic goiter) in autumn 2021 following severe bronchitis and subsequent hospitalization. She had a significant smoking history of approximately one pack of cigarettes per day, which is a well-established risk factor for severe TED progression. Her family history was negative for autoimmune and thyroid pathologies. In April 2022, despite treatment with methimazole (Tyrozol), total thyroidectomy was performed because of persistent thyroid disease and systemic autoimmune activity. Despite thyroidectomy, the clinical manifestations of TED continued to progress.

The clinical course of the disease was highly complicated by profound resistance to conventional conservative management. The patient had undergone multiple courses of intensive immunosuppressive therapy, including repeated cycles of systemic high-dose intravenous glucocorticosteroid pulse therapy, local retrobulbar injections of glucocorticoids, and cytostatic therapy. Due to the lack of clinically significant therapeutic response and the rapid progression of compressive orbital changes, the patient underwent a right-sided surgical orbital decompression in 2023, after which she completely ceased smoking.

During the current examination, severe ophthalmic deficits had remained prominent. Best-corrected visual acuity (BCVA) was reduced to 0.7 in the right eye (OD) and 0.5 in the left eye (OS), raising suspicion of early-stage compressive optic neuropathy. Intraocular pressure (IOP) was elevated to 27 mmHg OD and 26 mmHg OS, indicating secondary ocular hypertension likely caused by impaired venous outflow from the retrobulbar compartment. Symmetrical bilateral globe protrusion was confirmed by hertel exophthalmometry at 28 mm. Evaluation of the ocular adnexa showed pronounced bilateral upper eyelid retraction (13 mm), periorbital edema, lagophthalmos (incomplete eyelid closure), and noticeable conjunctival and caruncular injection, resulting in a Clinical Activity Score (CAS) of 6/7 and classifying her condition as severe, active TED according to the EUGOGO classification (Figure 1A; Table 1).

Laboratory profiling supported the persistence of systemic autoimmune activity. Testing revealed elevated levels of thyroid-stimulating hormone (TSH) at 4.57 mIU/L, anti-thyroid peroxidase (anti-TPO) antibodies at 12.5 IU/mL, and a marked elevation of thyroid-stimulating hormone receptor antibodies (TRAb) at 31.1 IU/L. Immunological analysis of peripheral blood demonstrated clear cellular immune dysregulation, characterized by a decreased CD4/CD8 ratio of 1.0, IgG hypergammaglobulinemia (25.72 g/L), and an eosinophil count of 8%. Furthermore, the functional phagocytic activity of the immune system was significantly suppressed, as indicated by the Nitroblue Tetrazolium (NBT) reduction test (spontaneous: 5%, induced: 8%).

Computed tomography (CT) of the orbits confirmed bilateral exophthalmos, diffuse enlargement of the extraocular muscles (with the most prominent changes observed in the medial recti), and increased orbital fat volume. A narrowing of the retrobulbar space was clearly visualized, consistent with active orbital inflammation, alongside signs of chronic tissue remodeling and fibrotic changes within the extraocular muscles (Figure 1B,C).

A crucial and defining aspect of this clinical case is that the patient’s TED continued to actively progress long after a total thyroidectomy. The persistence of severe inflammatory remodeling in the absence of thyroid tissue strongly supports the concept of thyroid-independent orbital autoimmune autonomy, indicating that localized orbital inflammation can be sustained independently of active thyroid antigen presentation.

### 3.2. Annotation of PBMC Cell Clusters

In total, after strict quality control and filtering, 5759 high-quality cellular transcriptomes were identified, including 4150 cells from the patient with thyroid eye disease (TED) and 1609 cells from the healthy donor. For the TED patient sample, the transcriptomes exhibited a median of 1999 genes per cell with a median UMI count of 5730, achieved at an average sequencing depth of 71,132 mean reads per cell. Simultaneously, the healthy control sample demonstrated a median of 1968 genes per cell and a median UMI count of 5329 per cell, with an average depth of 88,640 mean reads per cell.

**Figure 2:**
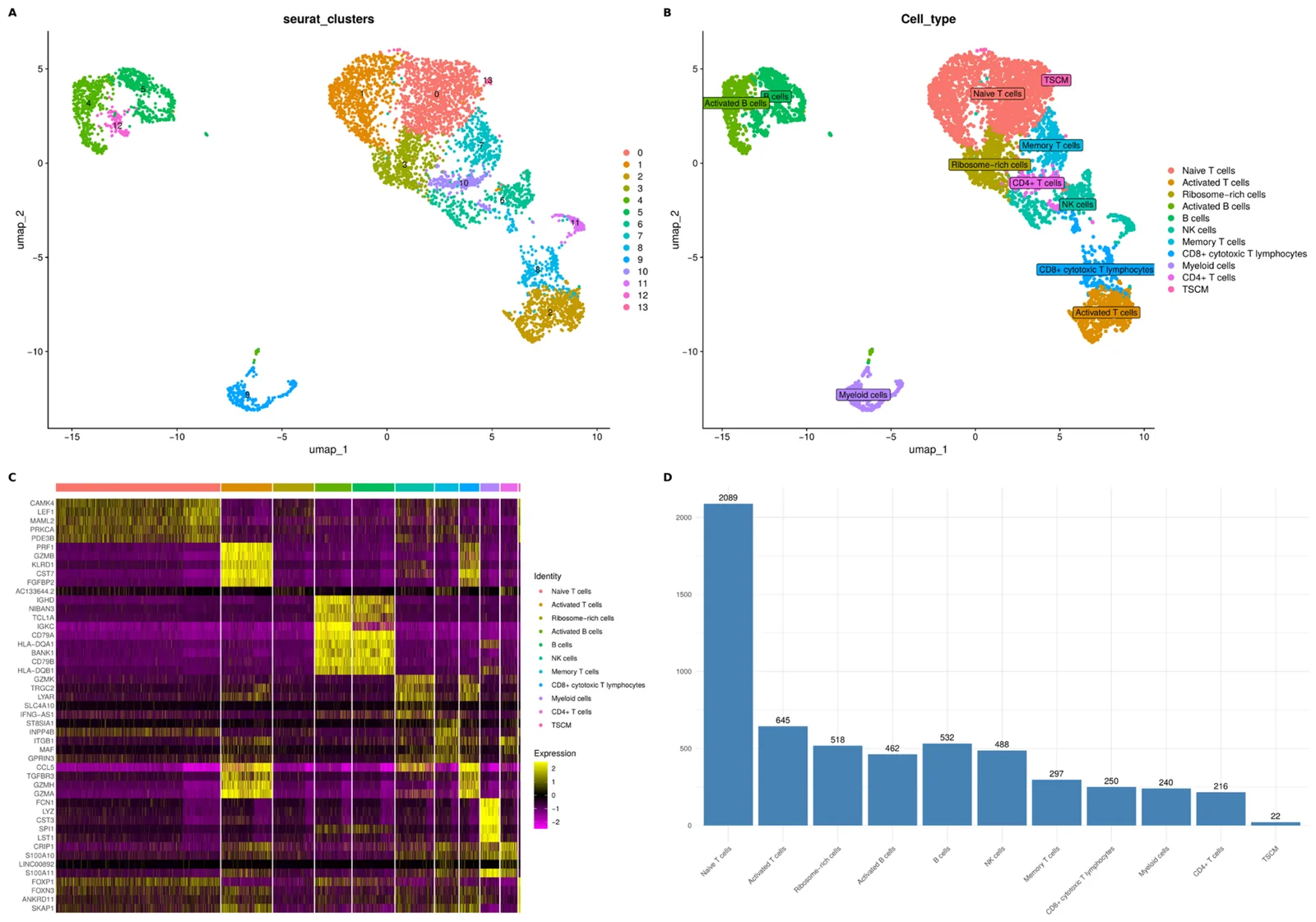
Single-cell transcriptomic landscape and composition of peripheral blood mononuclear cells (PBMCs) from the TED patient and the healthy donor. (**A**) Uniform Manifold Approximation and Projection (UMAP) visualization of 5759 high-quality single cells, color-coded by their respective unsupervised Seurat cluster assignments (0 to 13). (**B**) UMAP projection of the dataset with cells color-coded and labeled according to their final annotated cell type identities. (**C**) Expression heatmap of the top canonical marker genes across the identified cell populations. Rows represent individual marker genes (including *LEF1*, *GZMB*, *CD79A*, and *LYZ*), and columns represent single cells grouped by their annotated cell type identity. The scale bar indicates the normalized expression level (z-score). (**D**) Bar chart illustrating the absolute cell counts for each annotated cell type population across the merged single-cell dataset.

Cell clustering using the Louvain/Leiden algorithm and UMAP analysis led to the identification of 14 discrete cell clusters, numbered from 0 to 13 (Figure 2A). To define the specific cell subsets and establish their functional identities, we analyzed the cluster-specific differential gene expression profiles against a canonical set of lineage markers, visualized via a comprehensive expression heatmap (Figure 2C). The heatmap revealed highly distinct, clean, block-like expression patterns across the columns, confirming the distinct transcriptional boundaries between the identified leukocyte populations.

As shown in the heatmap (Figure 2C), the lymphoid and myeloid compartments exhibited mutually exclusive gene signatures. Naive T cells were clearly delineated by the enriched expression of *LEF1*. The cytotoxic cell pool encompassing NK cells and CD8^+^ cytotoxic T lymphocytes showed sharp, coordinated upregulation of cytolytic effector molecules and receptors, including *GZMB*, *GZMK*, *PRF1*, and *KLRD1*. The B-cell lineages (both B cells and Activated B cells) were characterized by a highly localized expression block of classical pan-B markers such as *CD79A*, *TCL1A*, and *BANK1*. In contrast, the myeloid cell compartment was uniquely distinguished from all lymphoid clusters by a prominent, dense expression cluster of canonical myeloid and monocytic genes, notably *LYZ*, *S100A8*, *S100A9*, and *S100A12*.

Based on these definitive transcriptomic signatures, the final annotated clusters were successfully projected onto the UMAP space (Figure 2B) and categorized into 11 distinct cell types: Naive T cells (2089 cells), Activated T cells (645 cells), Ribosome-rich cells (518 cells), Activated B cells (462 cells), B cells (532 cells), NK cells (488 cells), Memory T cells (297 cells), CD8^+^ cytotoxic T lymphocytes (250 cells), Myeloid cells (240 cells), CD4^+^ T cells (216 cells), and stem cell-like memory T cells (TSCM) (22 cells). Quantitative evaluation of the cell type distribution demonstrated that the Naive T-cell population constituted the largest component within the compiled PBMC dataset (Figure 2D). After this initial baseline annotation, each distinct cell type compartment was isolated for further high-resolution sub-clustering, characterization, and downstream functional pathway analysis.

### 3.3. Comparative Analysis of Immune Cell Composition and Transcriptomic Shifts in TED

**Figure 3:**
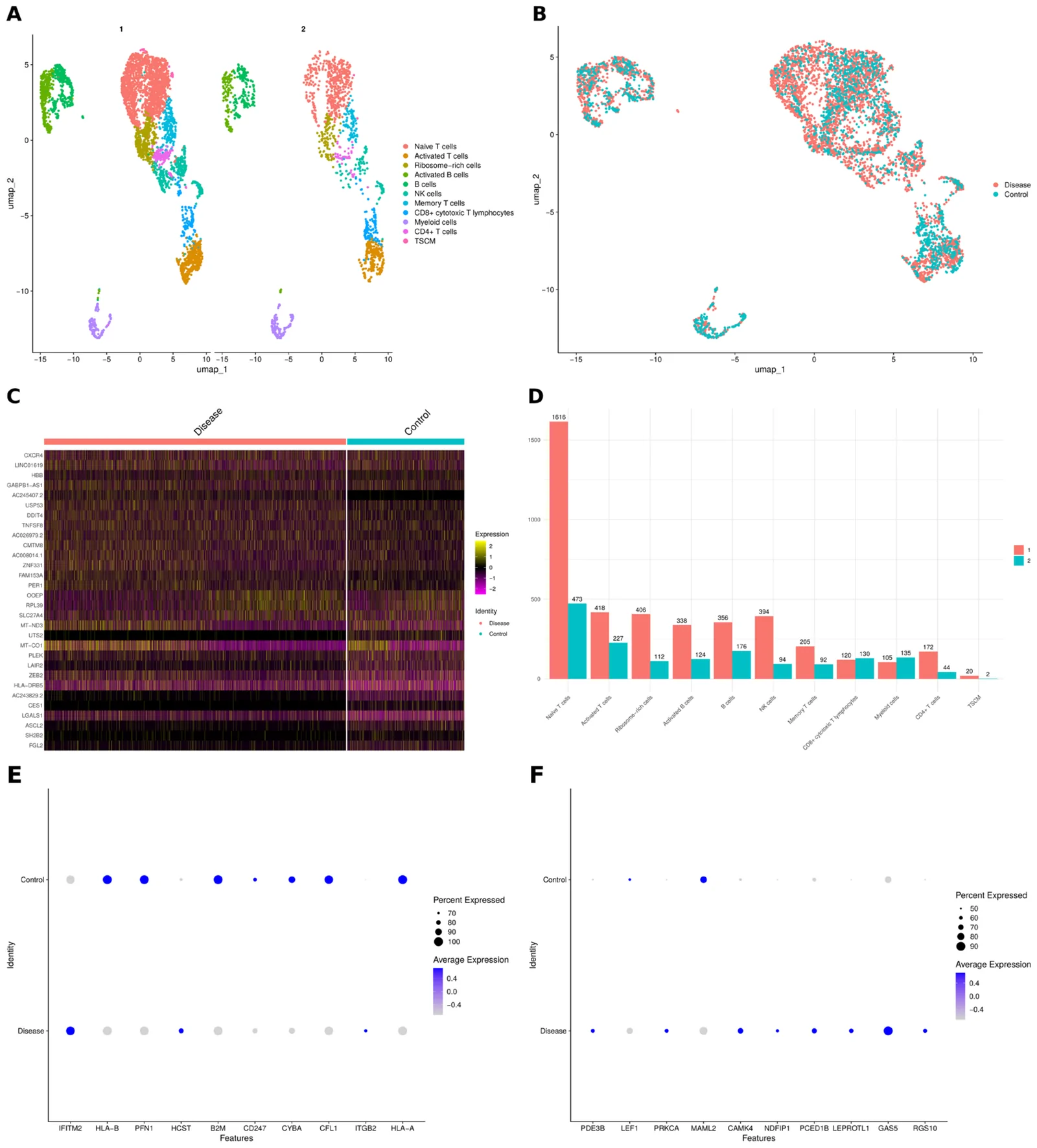
Comparative single-cell transcriptomic profiling of the peripheral immune landscape in TED and healthy control. (**A**) Uniform Manifold Approximation and Projection (UMAP) visualization of the discrete cell clusters split side-by-side by individual sample background (Sample 1 vs. Sample 2) to show topographic distribution shifts. (**B**) UMAP projection with cells from both samples overlaid on a single Harmony-integrated embedding, colored by sample identity (Disease vs. Control), demonstrating that cells are well mixed across shared clusters. (**C**) Comprehensive single-cell differential expression heatmap systematically contrasting the global transcriptomic landscapes between the Disease and Control conditions, with top pathogenic drivers (including *CXCR4*, *DDIT4*, *LINC01619*, and *TNFSF8*) highlighted along the y-axis. (**D**) Frequency bar chart displaying the absolute cellular counts for each annotated cell type, cross-compared between Sample 1 (Disease) and Sample 2 (Control) to highlight lymphoid expansion. (**E**) Dot plot of select differentially expressed genes (including *HLA-A*, *HLA-B*, *B2M*, *IFITM2*, *HCST*, *CD247*, and *ITGB2*), detailing both the percentage of expressing cells (dot size) and mean expression intensity (color scale), demonstrating enrichment primarily within the Control condition. (**F**) Dot plot of the top signature transcripts driving the pathology (including *GAS5*, *RGS10*, *LEF1*, *CAMK4*, *LEPROTL1*, *PDE3B*, *PRKCA*, *MAML2*, *NDFIP1*, and *PCED1B*), showcasing a distinct transcriptional shift and upregulation across the Disease sample.

To elucidate the systemic immune dysregulation underlying active, refractory thyroid eye disease (TED), a comparative analysis of cellular proportions and transcriptomic profiles was performed between the TED patient and the healthy donor. UMAP topologies, integrated using Harmony and visualized with cells from both samples overlaid on a shared embedding, demonstrated that discrete cellular clusters were well mixed across both conditions, indicating that observed differences are unlikely to be driven primarily by batch effects (Figure 3A,B). Quantifying the absolute cellular distribution across the identified lineages revealed distinct structural alterations in the peripheral blood mononuclear cell (PBMC) compartment of the TED patient (Figure 3D). The lymphoid compartment showed a pronounced numeric dominance in the patient group (Sample 1), particularly within the Naive T cell, Activated T cell, and Ribosome-rich cell clusters, highlighting a state of systemic immune mobilization. Conversely, the healthy donor sample (Sample 2) maintained a more compressed distribution, reflecting a baseline, non-inflammatory immune equilibrium (Figure 3D).

To define the molecular mechanisms driving this cellular reorganization, direct differential gene expression (DEG) analysis was conducted to compare the global transcriptomic matrices of the disease and control groups using the MAST algorithm (Log2 Fold Change > 0.5, adj. *p*-value < 0.05). The resulting expression profile revealed a clear transcriptional inversion between the two clinical states, as illustrated by a comprehensive single-cell heatmap (Figure 3C). Cells from the healthy donor (control) were defined by the sustained expression of major histocompatibility complex (MHC) structural elements, cellular architecture factors, and homeostatic regulators, including *HLA-A*, *HLA-B*, *B2M*, *PFN1*, *CYBA*, and *CFL1*, alongside cytotoxic/activation-associated markers such as *IFITM2*, *HCST*, *CD247*, and *ITGB2* (Figure 3C,E), as well as *MAML2* (Figure 3F).

Conversely, the TED patient’s transcriptomic landscape was profoundly dominated by a coordinated upregulation of pro-inflammatory mediators, cellular stress response elements, and homing receptors (Figure 3C). Among the top upregulated genes visualized in the heatmap, the chemokine receptor *CXCR4* and the metabolic/hypoxia stress regulator *DDIT4* exhibited extensive expression density across the patient’s cells, which may suggest an increased capacity for tissue homing and an active response to a high-stress microenvironment (Figure 3C). This was accompanied by a robust induction of immediate-early response transcription factors and downstream inflammatory drivers, including *FOS*, *JUNB*, *FOSB*, *TNFAIP3*, *GADD45A*, and *GADD45B*, which are critical mediators of immune activation and cellular survival pathways (Figure 3C). Furthermore, the disease state was characterized by the elevated expression of specialized non-coding regulatory elements and signaling modulators such as *LINC01619* and *TNFSF8* (Figure 3C), alongside a marked upregulation of *GAS5*, *RGS10*, *LEF1*, *CAMK4*, *LEPROTL1*, *PDE3B*, *PRKCA*, *NDFIP1*, *PCED1B*, and *MAML2* as shown by dot plot analysis (Figure 3F). Taken together, these granular transcriptomic shifts reveal a systemic transition from quiescent immune surveillance to an active, stress-induced inflammatory phenotype, mirroring the severe clinical manifestations observed in the patient’s orbital region.

### 3.4. Functional Enrichment and Pathway Analysis of TED-Specific Pathogenic Drivers

#### 3.4.1. Transcriptional Signatures and Signaling Polarization Within the T-Cell Compartment

**Figure 4:**
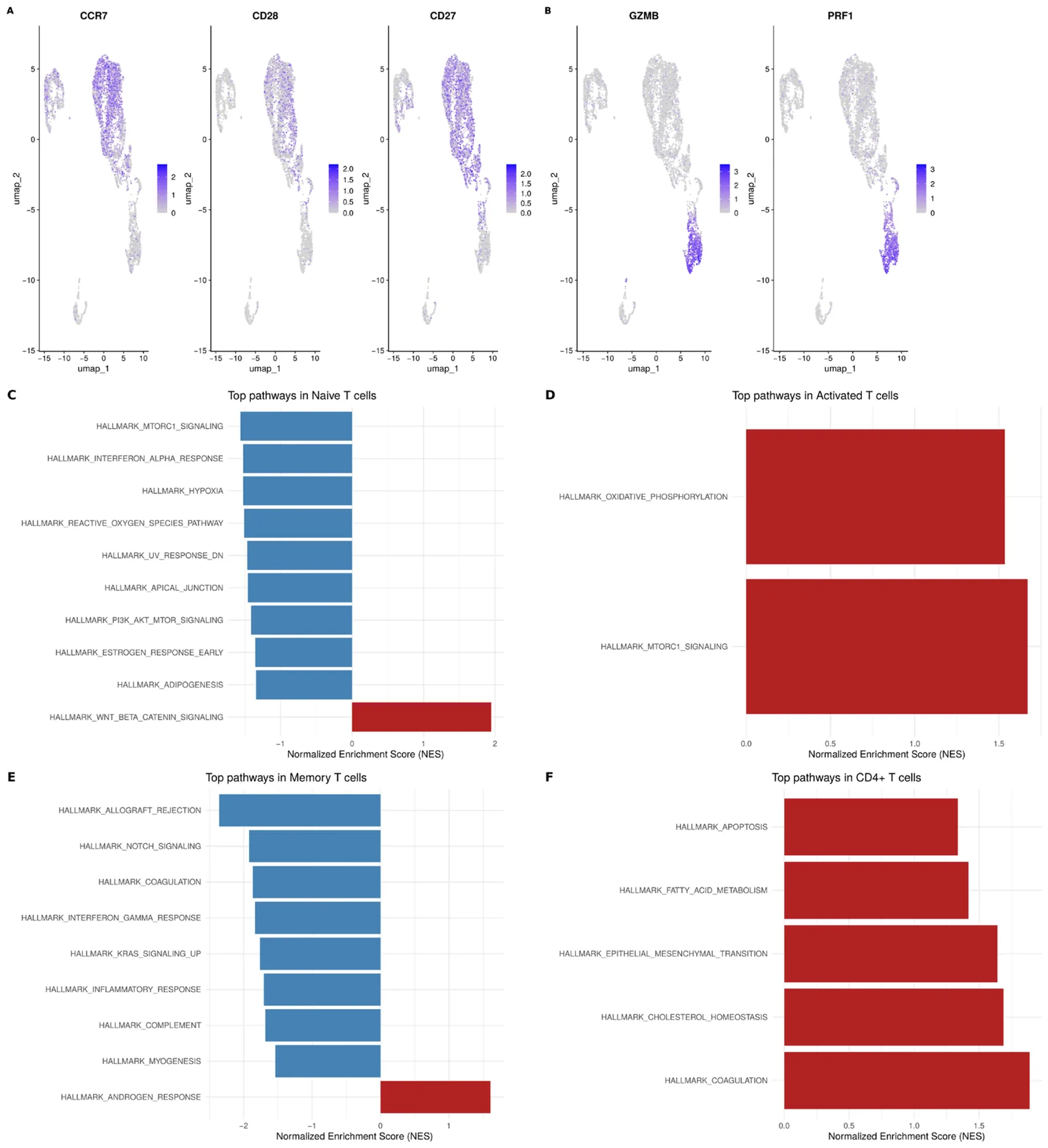
Dissection of T-cell compartment heterogeneity and functional polarization in TED. (**A**) UMAP feature plots illustrating the localized expression of canonical lymphoid homing and naive state markers (*CCR7*, *CD28*, and *CD27*). (**B**) UMAP feature plots demonstrating the expression of cytotoxic effector molecules (*GZMB* and *PRF1*) defining effector cell clusters. Color scales indicate normalized expression intensity. (**C**–**F**) Gene Set Enrichment Analysis (GSEA) bar charts highlighting the top significantly shifted Hallmark pathways across annotated T-cell subsets: (**C**) Naive T cells, (**D**) Activated T cells, (**E**) Memory T cells, and (**F**) CD4^+^ T cells. Red bars denote positive Normalized Enrichment Scores (NES) representing pathway activation, while blue bars denote negative NES values representing pathway suppression (adj. *p*-value < 0.05).

Analysis of the T-cell pool demonstrated clear lineage segregation based on the expression of canonical markers. Naive and central regulatory molecules, including *CCR7*, *CD28*, and *CD27*, localized predominantly to the lymphoid clusters on the UMAP projection (Figure 4A), whereas the cytotoxic effectors granzyme B (*GZMB*) and perforin (*PRF1*) strictly defined the active effector T lymphocyte and NK cell populations (Figure 4B).

Differential functional analysis via Gene Set Enrichment Analysis (GSEA) of these T-cell subsets revealed a mosaic pattern of pathological shifts. Naive T cells from the TED patient showed marked suppression of mTORC1 signaling, interferon-alpha response, hypoxia, and PI3K/AKT/mTOR pathways, which had occurred alongside a sharp upregulation of the Wnt/β-catenin signaling pathway (Figure 4C). This molecular profile may reflect adaptive transcriptomic modifications or altered differentiation dynamics within the naive T-cell pool under chronic inflammatory conditions.

Simultaneously, activated T cells underwent a classic hypermetabolic transformation, characterized by extremely high enrichment of oxidative phosphorylation and mTORC1 signaling cascades (Figure 4D). Memory T cells exhibited deep suppression of allograft rejection, Notch signaling, and coagulation pathways, contrasted by an isolated induction of androgen response signatures (Figure 4E). Finally, the CD4^+^ T cell subpopulation was uniquely characterized by the coordinated induction of apoptosis, fatty acid metabolism, cholesterol homeostasis, and epithelial–mesenchymal transition (EMT) programs (Figure 4F). This multi-pathway activation exposes the molecular substrate behind the heightened vulnerability of the helper T-cell arm and its predisposition to programmed cell death during severe TED.

#### 3.4.2. B-Cell Lineage Characterization and Compensatory Transcriptional Silencing

**Figure 5:**
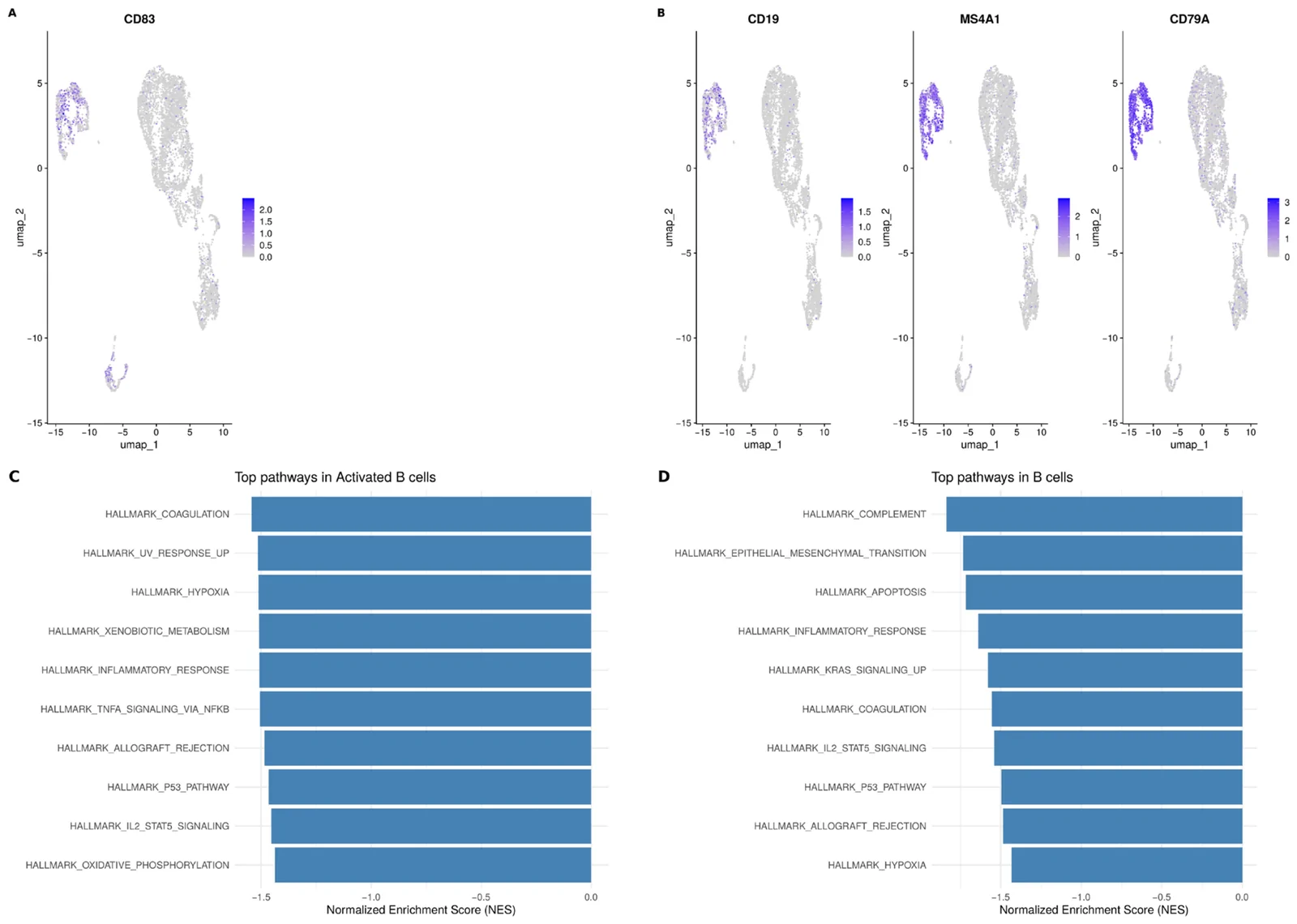
Characterization of B-cell lineage distribution and transcriptional activation patterns in TED. (**A**) UMAP feature plot showing the localized expression of the activation-associated marker *CD83*, demarcating the activated B-cell subpopulation. (**B**) UMAP feature plots displaying the expression profiles of canonical pan-B cell lineage markers (*CD19*, *MS4A1*, and *CD79A*), confirming cluster homogeneity. Color bars represent normalized expression intensity. (**C**) GSEA bar chart illustrating the top significantly suppressed Hallmark pathways in Activated B cells based on Normalized Enrichment Scores (NES). (**D**) GSEA bar chart showing the significantly suppressed Hallmark cascades within the general B-cell population. For both (**C**,**D**), blue bars indicate negative NES values, denoting uniform pathway suppression evaluated at a significance threshold of adj. *p*-value < 0.05.

To map the spatial distribution and activation state of the B-cell lineage within the peripheral blood mononuclear cell (PBMC) pool, we examined the expression of definitive lineage-specific markers. Expression of the early activation marker *CD83* was found to be tightly restricted to a specialized subpopulation corresponding to activated B cells (Figure 5A). Concurrently, the canonical pan-B cell marker transcripts *CD19*, *MS4A1* (CD20), and *CD79A* demonstrated robust, overlapping expression signals, revealing the precise topographic localization and high transcriptomic homogeneity of the mature B-lymphocyte pool within the UMAP embedding (Figure 5B).

Functional profiling via Gene Set Enrichment Analysis (GSEA) unveiled a coordinated downregulation of essential metabolic and signaling homeostatic programs across both identified B-cell compartments in the TED patient. Activated B cells were characterized by a uniform suppressive signature affecting critical homeostatic and activation pathways, including coagulation, UV response up, hypoxia, xenobiotic metabolism, inflammatory response, and TNF-α signaling via NF-κB (Figure 5C). Furthermore, this active cluster exhibited negative enrichment scores for allograft rejection, the p53 pathway, IL-2/STAT5 signaling, and oxidative phosphorylation, indicating a widespread suppression of both metabolic output and immune effector signaling (Figure 5C).

A highly convergent suppressive phenotype was observed within the general, steady-state B cell population (Figure 5D). GSEA revealed a deep negative regulation of the complement system, epithelial–mesenchymal transition, apoptosis, inflammatory response, and KRAS signaling, alongside a shared dampening of coagulation, IL-2/STAT5 signaling, the p53 pathway, allograft rejection, and hypoxia cascades (Figure 5D). This global down-regulation of metabolic and inflammatory pathways across both clusters points toward a state of profound compensatory transcriptional silencing or peripheral metabolic exhaustion in circulating B cells. This systemic phenotype is likely driven by the preferential homing and massive egress of highly active, pathogenic B-cell variants from the peripheral blood into the target orbital tissues during active TED, leaving a functionally depleted signature in the remaining circulating pool.

#### 3.4.3. Global Pathway Rewiring and Non-Lymphoid Immune Subpopulation Signatures

**Figure 6:**
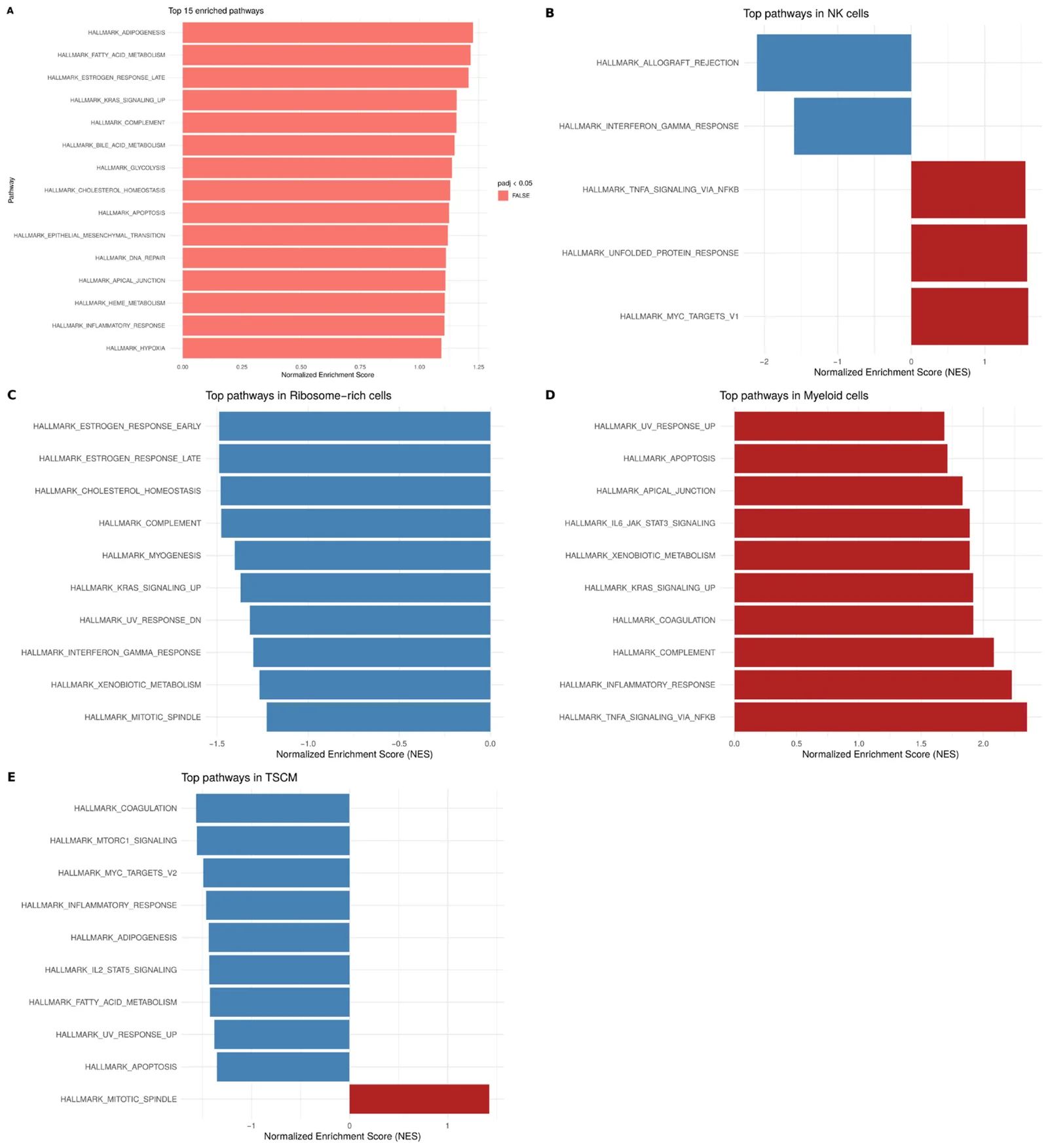
Global functional pathway enrichment and specialized non-lymphoid immune signatures in TED. (**A**) Overarching GSEA bar plot summarizing the Top 15 globally enriched Hallmark pathways across the entire single-cell dataset, ranked by positive Normalized Enrichment Scores (NES). (**B**) GSEA bar chart for the NK cell population, illustrating a bidirectional shift defined by the suppression of cytotoxic pathways (blue) and the activation of stress-induced inflammatory cascades (red). (**C**) GSEA bar chart deciphering the ribosome-rich cell cluster, demonstrating a uniform down-regulation across metabolic, structural, and signaling Hallmark gene sets. (**D**) GSEA bar chart for Myeloid cells, showing a robust, multi-pathway activation signature driving acute systemic inflammation. (**E**) GSEA bar chart for TSCM, showcasing comprehensive pathway dampening contrasted by the isolated upregulation of the mitotic spindle network. All pathways were evaluated at a significance threshold of adj. *p*-value < 0.05.

To systematically decode the global functional architecture and metabolic shifts occurring within total peripheral blood mononuclear cells (PBMCs) during active TED, an overarching Gene Set Enrichment Analysis (GSEA) was performed utilizing the comprehensive Hallmark signature collection. A global evaluation of the top 15 enriched cascades across the entire dataset revealed a uniform, positive activation pattern highlighting systemic metabolic and signaling rewiring (Figure 6A). This baseline landscape was heavily driven by the up-regulation of adipogenesis, fatty acid metabolism, late estrogen response, KRAS signaling, and complement cascades, alongside vital survival and structural pathways such as glycolysis, cholesterol homeostasis, apoptosis, epithelial–mesenchymal transition (EMT), and hypoxia response (Figure 6A).

Detailed dissection of these processes at the single-cell lineage level revealed distinct, heterogeneous regulatory patterns among non-lymphoid and specialized cell populations. The NK cell population demonstrated a complex, bidirectional regulatory phenotype (Figure 6B). Classical cytotoxic effector tracks, including allograft rejection and interferon-gamma responses, were markedly suppressed, whereas cellular stress and pro-inflammatory pathways—specifically TNF-α signaling via NF-κB, the unfolded protein response (UPR), and MYC targets v1—were concurrently induced (Figure 6B). This divergent pattern reflects a dissociation between NK cell cytotoxic effector programs and stress/inflammatory signaling: interferon-gamma response and allograft rejection, pathways closely linked to cytotoxic function, were suppressed, while pathways reflecting cellular stress and pro-inflammatory activation were induced. This signature points toward a state of functional exhaustion affecting classical cytotoxic responses, coupled with a persistent, stress-induced inflammatory phenotype.

A highly coordinated suppressive phenotype was observed within the ribosome-rich cell cluster (Figure 6C). This subpopulation demonstrated an all-negative enrichment profile across key signaling and metabolic axes, exhibiting pronounced downregulation of early and late estrogen responses, cholesterol homeostasis, complement cascades, myogenesis, KRAS signaling, UV response, interferon-gamma response, xenobiotic metabolism, and mitotic spindle networks (Figure 6C).

By contrast, the patient’s myeloid cells exhibited a robust, uniform signature of acute inflammatory activation, with all top Hallmark pathways showing strong positive enrichment (Figure 6D). This pathogenic myeloid profile was driven by elevated scores for UV response up, apoptosis, apical junction maintenance, and IL-6/JAK/STAT3 signaling, alongside metabolic and inflammatory cascades including xenobiotic metabolism, KRAS signaling up, coagulation, complement system, inflammatory response, and TNF-α signaling via NF-κB (Figure 6D). This extensive activation profile supports a potential role of myeloid cells in sustaining and amplifying systemic inflammation in TED.

Finally, TSCM largely mirrored a suppressive homeostatic profile, showing negative enrichment for coagulation, mTORC1 signaling, MYC targets v2, inflammatory response, adipogenesis, IL-2/STAT5 signaling, fatty acid metabolism, UV response up, and apoptosis (Figure 6E). Crucially, the TSCM pool exhibited an isolated induction of the mitotic spindle network, which may be consistent with partial preservation of proliferative capacity, although mitotic spindle enrichment alone does not directly demonstrate long-term self-renewal potential and would require functional validation (e.g., proliferation or clonogenic assays) (Figure 6E).

## 4. Discussion

Thyroid eye disease (TED) is a severe, organ-specific autoimmune disorder of the orbit whose management remains a formidable clinical challenge, especially when characterized by absolute refractoriness to conventional glucocorticoid pulse therapy and cytostatic regimens. In this report, we present an unusual clinical scenario of a progressive TED presentation where aggressive retrobulbar tissue inflammation persisted and advanced despite a total thyroidectomy performed in 2022. This clinical course directly challenges the traditional paradigm asserting that complete ablation of the thyroid gland and subsequent removal of shared thyroid-orbital antigens guarantees orbitopathy resolution [18]. Rather than demonstrating complete orbital immune autonomy, the present case suggests that the immune processes sustaining TED can persist after thyroidectomy and may become at least partially uncoupled from the continued presence of thyroid tissue. By conducting single-cell RNA sequencing (scRNA-seq) of peripheral blood mononuclear cells (PBMCs) in this patient, we sought to characterize the systemic immune-cell states accompanying this unusual and refractory clinical phenotype.

At the whole-dataset level, global gene set enrichment analysis (GSEA) revealed a substantial transcriptional remodeling of the patient’s immune cell metabolism, highlighted by xenobiotic, fatty acid, and glycolytic pathways, intertwined with the activation of hypoxia and apoptosis. Such transcriptomic shifts are highly characteristic of circulating immune components exposed to sustained inflammatory and oxidative stress [19]. Importantly, however, the observed immune remodeling was not uniform across all cell populations. Instead, individual immune compartments displayed distinct and sometimes opposing transcriptional states, suggesting that persistent TED may involve heterogeneous immune-cell responses rather than generalized activation of the entire circulating immune system. The most aggressive pro-inflammatory phenotype was identified within the myeloid compartment, which showed strong enrichment of TNF- α signaling via NF- κB and IL-6/JAK/STAT3 cascades. This peripheral inflammatory phenotype is broadly compatible with the inflammatory cellular landscapes previously described in orbital tissues from patients with active TED [13]. Given that IL-17 can promote inflammatory signaling through NF-κB-associated pathways, these observations are also consistent with our previous serum- and tear-based findings in an independent TED cohort, in which active disease was associated with elevated IL-17 and reduced IL-38 levels, with serum IL-17 correlating with the Clinical Activity Score (r = 0.885, *p* = 0.001) [20]. Because these cytokine measurements were obtained from a separate cohort, they should be considered supportive biological context rather than direct validation of the present single-cell findings. Nevertheless, the convergence of these observations supports further investigation of interconnected cytokine networks in persistent TED. Characterization of the T-cell compartment revealed substantial functional heterogeneity. Activated T cells demonstrated strong enrichment of oxidative phosphorylation and mTORC1 signaling, pathways associated with the increased metabolic requirements of activated immune cells. This metabolic phenotype may reflect increased bioenergetic demand associated with T-cell activation, although the present data cannot establish whether these cells are directly pathogenic to orbital tissues. In contrast, naive T cells showed suppression of several mTOR-related and inflammatory pathways together with enrichment of Wnt/β-catenin signaling, potentially reflecting altered differentiation or maintenance of the naive T-cell compartment under chronic inflammatory conditions. Furthermore, the cytotoxic CD8+ T-lymphocyte pool exhibited marked expression of granzyme B (GZMB) and perforin 1 (PRF1), consistent with the presence of circulating cytotoxic lymphocytes. However, expression of cytotoxic markers alone does not demonstrate that these cells recognize or damage orbital tissues, and functional studies would be required to establish their contribution to TED pathology.

A major and counterintuitive finding of our study was the global downregulation of classical inflammatory response, complement, and oxidative phosphorylation hallmarks within circulating B lymphocytes (including both canonical *CD19*^+^*MS4A1*^+^*CD79A*^+^ and activated *CD83*^+^ clusters) and NK cells. While unexpected during active autoimmune disease, this pattern highlights the complexity of the circulating immune compartment. One possible explanation is that the peripheral blood represents only a transient snapshot of immune-cell states during active tissue inflammation. Activated lymphocyte populations may undergo redistribution from the circulation toward inflamed tissues, potentially resulting in a relative depletion of highly activated cells from the peripheral blood. Under this model, activated B-cell and NK-cell populations could preferentially migrate toward inflamed extraocular muscles and retrobulbar adipose tissue along chemokine gradients [21]. However, this “tissue-drainage” or immune-redistribution hypothesis cannot be directly demonstrated by the present study because orbital tissue was not analyzed and cell migration was not experimentally measured. Alternative explanations, including treatment-related effects, donor-specific variation, cellular exhaustion, or compensatory transcriptional regulation, must therefore also be considered [22].

The upregulation of the chemokine receptor CXCR4 and the metabolic/stress regulator DDIT4 provides a potentially relevant molecular framework for interpreting these observations. Elevated CXCR4 expression may increase the capacity of circulating immune cells to respond to CXCL12-dependent tissue-homing signals [23]. Concurrently, *DDIT4* serves as a critical cell-preservation hub under inflammatory hypoxia, inhibiting excessive mTORC1 activation to prevent premature cell death in hostile environments. This is supported by the parallel induction of immediate-early response factors (*FOS*, *JUNB*, *FOSB*) and the NF-κB feedback regulator *TNFAIP3*, suggesting that the circulating immune pool may exist in a state of advanced molecular priming, potentially poised to execute pro-inflammatory programs upon tissue entry.

These systemic findings allow us to hypothesize that targeting the *CXCR4*/*CXCL12* axis could disrupt the pathogenic homing cascade, while modulating cell-stress pathways may help overcome glucocorticoid resistance in severe TED; however, this remains a hypothesis derived from a single case and requires validation at the protein and functional level before any therapeutic implications can be drawn.

The persistent activation of TNF-α/NF-κB signaling observed across multiple immune populations in this patient may offer a candidate mechanistic explanation for her documented clinical resistance to glucocorticoid pulse therapy: NF-κB is known to compete with the glucocorticoid receptor for shared transcriptional coactivators such as p300, thereby limiting glucocorticoid-driven anti-inflammatory gene transcription [24]. The concurrent upregulation of TNFAIP3 (A20), a negative feedback regulator of NF-κB previously implicated in polyautoimmune syndromes that include autoimmune thyroiditis [25], may reflect an insufficient compensatory attempt to restrain this inflammatory signaling.

These findings carry potential clinical relevance. The IL-6/JAK/STAT3 axis, activated within the myeloid compartment of this patient, is targeted by tocilizumab, an IL-6 receptor inhibitor already used as second-line therapy for corticosteroid-resistant TED, with recent meta-analyses confirming its efficacy in reducing proptosis and clinical activity scores [26,27]. The enrichment of IL-6/JAK/STAT3 signaling in the present patient is therefore consistent with a pathway already implicated in the treatment of refractory TED. However, the present single-patient transcriptomic profile cannot predict treatment response or establish that pathway-directed therapy would be effective in this individual. Downstream of this same signaling network, JAK inhibitors such as upadacitinib—already approved for rheumatoid arthritis, a disease sharing overlapping TNF-α/NF-κB and IL-6/JAK/STAT3 inflammatory pathways [28]—may represent an area for future investigation in TED. Any consideration of JAK inhibition in TED should currently remain exploratory and should be evaluated in appropriately designed preclinical or clinical studies rather than inferred as a treatment recommendation from this single case.

Overall, the present findings support a model in which persistent TED after thyroidectomy may involve continued systemic immune remodeling that is not completely dependent on the presence of thyroid tissue. The peripheral immune landscape was characterized by inflammatory activation of myeloid cells, metabolic reprogramming of activated T cells, stress-associated transcriptional responses, and comparatively suppressed inflammatory programs in circulating B-cell and NK-cell populations. The coexistence of these divergent states suggests that persistent TED may reflect dynamic redistribution and functional specialization of immune-cell populations rather than a uniformly activated systemic immune response. In particular, CXCR4-associated signals and DDIT4-associated stress responses provide testable hypotheses linking circulating immune-cell states with the persistence of tissue inflammation.

Importantly, this study should be interpreted as a hypothesis-generating molecular case study rather than evidence of a generalizable TED transcriptomic signature. The comparison involves one patient and one healthy donor, therefore, the large number of profiled cells does not constitute biological replication. Patient-specific factors, including previous smoking exposure, extensive immunosuppressive treatment, thyroid hormone status, and other clinical characteristics, may have contributed to the observed transcriptional differences. Furthermore, peripheral blood cannot directly reproduce the cellular and molecular environment of the orbit. The absence of paired orbital tissue, protein-level validation, and functional migration or signaling assays limits mechanistic inference. Future studies incorporating larger cohorts, longitudinal sampling, paired peripheral blood and orbital tissue, flow cytometry, protein-based measurements, and functional CXCR4/CXCL12 and NF-κB/glucocorticoid-receptor assays will be necessary to determine whether the signatures identified here represent reproducible features of persistent or refractory TED.

## Figures and Tables

**Table 1:** Clinical, ophthalmological, and laboratory characteristics of the patient with progressive, refractory thyroid eye disease (TED).

Parameter	Value
Demographics & Clinical History	
Sex	Female
Age	47 years
Primary Diagnosis	Graves’ disease with severe active thyroid eye disease (TED)
Year of Graves’ disease diagnosis	2021
Total thyroidectomy (Year)	April 2022
Disease course after thyroidectomy	Progressive TED despite thyroidectomy
Smoking history	~1 pack/day; smoking cessation after orbital decompression in 2023
Family history	Negative for autoimmune and thyroid diseases
Previous treatment	Methimazole, repeated intravenous glucocorticoid pulse therapy, retrobulbar glucocorticoids, cytostatic therapy
Treatment response	Refractory
Prior orbital surgery	Right orbital decompression (2023)
Ophthalmic Assessment & Severity	
Clinical Activity Score (CAS)	6/7
European Group on Graves’ Orbitopathy (EUGOGO) severity	Severe active TED
Best-corrected visual acuity (BCVA)	OD: 0.7; OS: 0.5
Intraocular pressure (IOP)	OD: 27 mmHg; OS: 26 mmHg
Hertel exophthalmometry	28 mm bilaterally
Upper eyelid retraction	13 mm bilaterally
Lagophthalmos	Present
Suspected compressive optic neuropathy	Early stage
Laboratory & Immunological Parameters	
TSH	4.57 mIU/L
Anti-TPO antibodies	12.5 IU/mL
TRAb	31.1 IU/L
CD4/CD8 ratio	1.0
Total IgG	25.72 g/L
Eosinophils	8%
NBT (Nitroblue Tetrazolium) test	Spontaneous: 5%; Induced: 8%
Imaging Findings	
Orbital CT	Bilateral exophthalmos, enlargement of extraocular muscles, increased orbital fat volume, narrowing of the retrobulbar space, inflammatory and fibrotic remodeling

## Data Availability

The original contributions presented in this study are included in the article. Further inquiries can be directed to the corresponding author.
